# Supplementary material for: Trade-Off Between Enzymatic Antioxidant Defense and Accumulation of Organic Metabolite Affects Salt Tolerance of White Clover Associated with Redox, Water, and Metabolic Homeostases
Source: Plants (Basel). 2025 Jan 7;14(2):145. doi: 10.3390/plants14020145 (PMC11768267; doi:10.3390/plants14020145)
Supplement: Supplementary file 1 [file plants-14-00145-s001.zip › plants-3364818-supplementary.pdf]

Table S1 Effects of cultivar, stress treatment, and their interactions on all parameters were analyzed by using two-way ANOVA. The "***" indicates $p < 0.001$.

Parameter	Item	Quadratic sum	Mean square	*F*	*p*
Chl a content	Cultivar	137.79	137.79	38.31	0.000***
	Treatment	27.64	27.64	7.68	0.024
	Cultivar&Treatment	21.43	21.43	5.96	0.040
Chl b content	Cultivar	0.53	0.53	6.17	0.038
	Treatment	28.42	28.42	328.80	0.000***
	Cultivar&Treatment	7.40	7.40	85.65	0.000***
Chl a/b	Cultivar	5.75	5.75	39.06	0.000***
	Treatment	0.73	0.73	4.96	0.056
	Cultivar&Treatment	0.15	0.15	1.03	0.340
Total chl content	Cultivar	111.54	111.54	25.03	0.001
	Treatment	210.01	210.01	47.13	0.000***
	Cultivar&Treatment	21.65	21.65	4.86	0.059
Relative water content	Cultivar	239.83	239.83	35.10	0.000***
	Treatment	627.21	627.21	91.80	0.000***
	Cultivar&Treatment	261.86	261.86	38.33	0.000***
Omostic potential	Cultivar	0.33	0.33	203.04	0.000***
	Treatment	1.59	1.59	985.50	0.000***
	Cultivar&Treatment	0.29	0.29	179.44	0.000***
Electrolyte leakage	Cultivar	70.78	70.78	8.74	0.018
	Treatment	2663.92	2663.92	329.01	0.000***
	Cultivar&Treatment	120.00	120.00	14.82	0.005
MDA content	Cultivar	42.75	42.75	37.06	0.000***
	Treatment	93.58	93.58	81.12	0.000***
	Cultivar&Treatment	65.30	65.30	56.60	0.000***
H_2O_2 content	Cultivar	20.15	20.15	19.15	0.002
	Treatment	30.29	30.29	28.79	0.001
	Cultivar&Treatment	13.74	13.74	13.06	0.007
$O_2^{\cdot-}$ content	Cultivar	0.05	0.05	3.47	0.100
	Treatment	0.82	0.82	59.59	0.000***
	Cultivar&Treatment	0.10	0.10	7.41	0.026
SOD activity	Cultivar	23776.98	23776.98	138.02	0.000***
	Treatment	26254.52	26254.52	152.40	0.000***
	Cultivar&Treatment	29182.12	29182.12	169.40	0.000***
POD activity	Cultivar	4243504.38	4243504.38	207.80	0.000***
	Treatment	84407.91	84407.91	4.13	0.076
	Cultivar&Treatment	3974708.60	3974708.60	194.64	0.000***
CAT activity	Cultivar	568.37	568.37	27.08	0.001
	Treatment	544.18	544.18	25.93	0.001
	Cultivar&Treatment	423.41	423.41	20.17	0.002
APX activity	Cultivar	493.40	493.40	20.19	0.002
	Treatment	520.59	520.59	21.30	0.002
	Cultivar&Treatment	780.26	780.26	31.92	0.000***
MDHR activity	Cultivar	0.68	0.68	2.34	0.164
	Treatment	10.62	10.62	36.35	0.000***
	Cultivar&Treatment	21.58	21.58	73.85	0.000***
DHAR activity	Cultivar	1103.01	1103.01	29.67	0.001
	Treatment	1256.03	1256.03	33.79	0.000***
	Cultivar&Treatment	2326.61	2326.61	62.59	0.000***
GR activity	Cultivar	1.56	1.56	37.11	0.000***
	Treatment	4.31	4.31	102.55	0.000***
	Cultivar&Treatment	1.30	1.30	31.02	0.001
ASA content	Cultivar	4.88	4.88	55.89	0.000***
	Treatment	3.96	3.96	45.38	0.000***
	Cultivar&Treatment	1.51	1.51	17.35	0.003
DHA content	Cultivar	0.02	0.02	55.87	0.000***

	Treatment	0.04	0.04	146.21	0.000***
	Cultivar&Treatment	0.01	0.01	18.51	0.003
ASA/DHA	Cultivar	659.94	659.94	12.81	0.007
	Treatment	1972.50	1972.50	38.30	0.000***
	Cultivar&Treatment	614.63	614.63	11.93	0.009
GSH content	Cultivar	0.00007	0.00007	26.44	0.001
	Treatment	0.00004	0.00004	15.94	0.004
	Cultivar&Treatment	0.00013	0.00013	50.00	0.000***
GSSG content	Cultivar	0.40	0.40	5.46	0.048
	Treatment	2.26	2.26	30.94	0.001
	Cultivar&Treatment	2.83	2.83	38.72	0.000***
GSH/GSSG	Cultivar	0.00	0.00	5.13	0.053
	Treatment	0.00	0.00	7.04	0.029
	Cultivar&Treatment	0.00	0.00	10.41	0.012
Glycolic acid	Cultivar	60.78	60.78	52.47	0.000***
	Treatment	15.75	15.75	13.60	0.006
	Cultivar&Treatment	0.74	0.74	0.64	0.446
Succinic acid	Cultivar	68.02	68.02	46.73	0.000***
	Treatment	188.21	188.21	129.31	0.000***
	Cultivar&Treatment	242.97	242.97	166.94	0.000***
Fumaric acid	Cultivar	26.14	26.14	4.88	0.058
	Treatment	360.30	360.30	67.32	0.000***
	Cultivar&Treatment	12.76	12.76	2.38	0.161
Malic acid	Cultivar	208.05	208.05	383.87	0.000***
	Treatment	65.14	65.14	120.19	0.000***
	Cultivar&Treatment	158.73	158.73	292.87	0.000***
Linolenic acid	Cultivar	9.14	9.14	19.37	0.002
	Treatment	4.50	4.50	9.54	0.015
	Cultivar&Treatment	2.52	2.52	5.34	0.050
Cis-sinapinic acid	Cultivar	0.22	0.22	45.55	0.000***
	Treatment	3.79	3.79	769.12	0.000***
	Cultivar&Treatment	0.13	0.13	26.60	0.001
Serine	Cultivar	228.94	228.94	2.44	0.157
	Treatment	1090.72	1090.72	11.62	0.009
	Cultivar&Treatment	573.84	573.84	6.11	0.039
Allothreonine	Cultivar	58.38	58.38	2.37	0.162
	Treatment	874.21	874.21	35.54	0.000***
	Cultivar&Treatment	452.31	452.31	18.39	0.003
4-Aminobutyric acid	Cultivar	134.39	134.39	38.30	0.000***
	Treatment	6.09	6.09	1.73	0.224
	Cultivar&Treatment	304.50	304.50	86.78	0.000***
Tagatose	Cultivar	16.96	16.96	2.13	0.183
	Treatment	1533.84	1533.84	192.26	0.000***
	Cultivar&Treatment	98.61	98.61	12.36	0.008
Fructose acid	Cultivar	0.00	0.00	0.00	0.995
	Treatment	148.75	148.75	132.79	0.000***
	Cultivar&Treatment	12.73	12.73	11.36	0.010
Glucoheptose	Cultivar	6.85	6.85	6.26	0.037
	Treatment	34.36	34.36	31.40	0.001
	Cultivar&Treatment	21.95	21.95	20.06	0.002
Cellobiose	Cultivar	0.99	0.99	542.70	0.000***
	Treatment	1.01	1.01	551.31	0.000***
	Cultivar&Treatment	0.65	0.65	354.75	0.000***
Melezitose	Cultivar	0.01	0.01	116.97	0.000***
	Treatment	0.01	0.01	104.56	0.000***
	Cultivar&Treatment	0.00	0.00	17.24	0.003***
Myo-inositol	Cultivar	114.33	114.33	35.54	0.000***
	Treatment	174.04	174.04	54.10	0.000***
	Cultivar&Treatment	84.52	84.52	26.27	0.001
Arabitol	Cultivar	51.57	51.57	189.55	0.000***
	Treatment	11.21	11.21	41.19	0.000***

	Cultivar&Treatment	0.02	0.02	0.06	0.816
	Cultivar	13.65	13.65	0.02	0.898
Galactinol	Treatment	519.10	519.10	0.67	0.438
	Cultivar&Treatment	29046.68	29046.68	37.25	0.000***
	Cultivar	60.65	60.65	435.87	0.000***
Cellobiotol	Treatment	15.20	15.20	109.22	0.000***
	Cultivar&Treatment	13.85	13.85	99.54	0.000***
	Cultivar	0.03	0.03	71.28	0.000***
Stigmasterol	Treatment	0.03	0.03	70.73	0.000***
	Cultivar&Treatment	0.01	0.01	31.34	0.001
